# Alpha-1-antitrypsin deficient man presenting with lung function decline associated with dust exposure: a case report

**DOI:** 10.1186/1752-1947-5-154

**Published:** 2011-04-19

**Authors:** Moshe Zutler, Patricia J Quinlan, Paul D Blanc

**Affiliations:** 1 505 Parnassus Avenue, M1097, San Francisco, CA 94143, USA; 2 1001 Potrero Avenue, San Francisco, CA 94110, USA; 3 350 Parnassus Avenue, Suite 609, San Francisco, CA USA

## Abstract

**Introduction:**

People with α_1_-antitrypsin deficiency are at increased risk for the development of chronic obstructive pulmonary disease. Previous retrospective epidemiologic studies have found that exposure to occupational dust among those with α_1_-antitrypsin deficiency is a risk factor at the group level for poorer lung function, but on an individual clinical basis, a causal attribution can be difficult to establish.

**Case presentation:**

We describe the case of a 68-year-old Caucasian man with a 25 pack-year smoking history who presented with new-onset dyspnea on exertion in the setting of workplace dust exposure. During his evaluation, he was found to have α_1_-antitrypsin deficiency with evidence of development of pulmonary emphysema. Workplace spirometric monitoring over 10 years of surveillance for an on-the-job respirator fit program demonstrated a sharp downward slope in forced expiratory volume in one second, or FEV_1_, during his periods of most significant dust exposure, which was attenuated after discontinuation of his workplace exposure.

**Conclusion:**

Patients with α_1_-antitrypsin disease should be assessed for occupational exposures and closely monitored for work-accelerated progression of lung function decline. More generally, this case report supports the biological plausibility of occupationally associated chronic obstructive pulmonary disease, underscoring that work-associated pulmonary disease can be multi-factorial.

## Introduction

On a population level, it is clear that occupational exposure in dusty trades are associated with the development of chronic obstructive pulmonary disease (COPD) in healthy individuals without other risk factors [1,2]. On an individual clinical basis, however, it is difficult to establish a direct link between a given occupational exposure and COPD. This even holds true in α_1_-antitrypsin (A1AT) deficiency, a genetic disorder that predisposes individuals to the development of pulmonary emphysema and supports the biological plausibility of dust-associated COPD [3-5]. We present a case of work-related COPD in A1AT deficiency in which attribution was aided by longitudinal lung function data collected prospectively over a protracted period of occupational exposure as well as through later follow-up.

### Case presentation

A 68-year-old Caucasian man presented with an initial complaint of dyspnea on exertion that had developed five years prior and had progressed to shortness of breath while walking up one flight of stairs. He was a former cigarette smoker from ages 14 to 30 years, with a maximum of one and a half packs per day (less than 25 pack-years total) and no reported respiratory symptoms during that period. He experienced significant inorganic dust exposure while grinding large concrete aquarium exhibition tanks over a period of 10 years, ending six years prior to presentation. Less dusty work exposure continued until his retirement at age 67. He had no other significant exposure to dusts or fumes.

His medical history was notable only for a prior traumatic cervical spine injury. His physical examination revealed pulmonary auscultation that was remarkable for a prolonged expiratory phase without wheezes or rhonchi. The remainder of his physical examination was otherwise unremarkable, except for a left-sided paresis (most notable in the upper extremity) consistent with his prior spinal injury.

**Figure 1:**
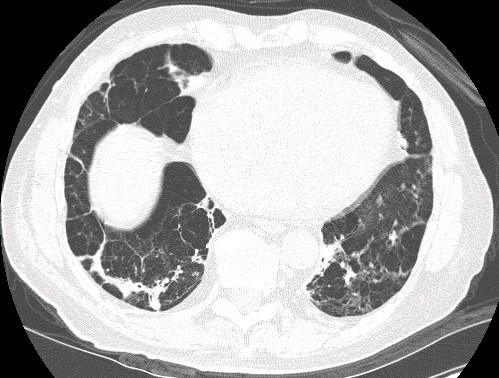
**Single computed tomography image of the patient's chest**. Bilateral lower-lobe emphysema with bullae is present, consistent with panlobular emphysema.

Pulmonary function testing at presentation demonstrated airflow obstruction: forced expiratory volume in one second (FEV_1_) was 2.10 litres (61% of the predicted value), forced vital capacity (FVC) was 3.60 litres (81% of the predicted value), the FEV_1_:FVC ratio was 0.58, forced expiratory flow over 25% to 75% of the expired volume (FEF_25-75_) was 0.85 litres per second (28% of the predicted value), total lung capacity (TLC) measured by plethysmography was 6.40 litres (93% of the predicted value), and residual lung volume (RV) was 2.91 litres (123% of the predicted value). There was minimal response to an inhaled bronchodilator. The diffusing capacity of the lung for carbon monoxide (D_L_CO) (hemoglobin-adjusted) was 18.5 ml/min/mmHg (59% of the predicted value), and the D_L_CO adjusted for alveolar volume (VA) was 3.2 (69% of the predicted value). A computed tomography scan of the chest demonstrated bilateral lower-lobe-predominant emphysema (Figure 1). A serum A1AT assay (electrophoresis with agarose immunofixation) indicated a PiZZ phenotype, with a quantified value of 24 U (normal ≥ 90 U). Treatment was initiated with a combination of inhaled fluticasone and salmeterol twice daily, tiotropium once daily and albuterol as needed.

**Figure 2:**
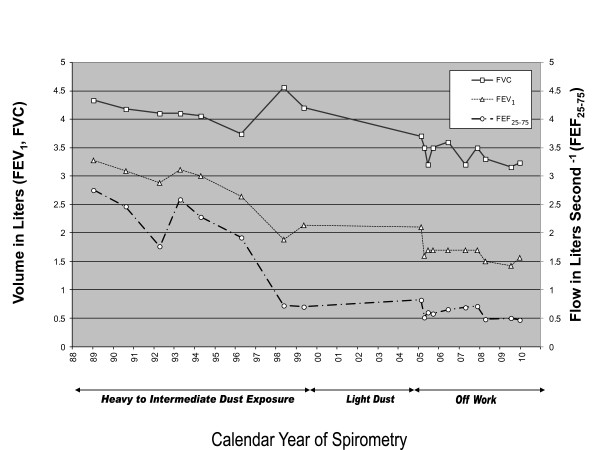
**Changes in forced expiratory volume in one second (FEV_1_), forced vital capacity (FVC) and forced expiratory flow over 25% to 75% of the expired volume (FEF_25-75_) over time during periods of heavy workplace dust exposure and light dust exposure as well as following work discontinuation**. Solid line, FVC; dotted line, FEV_1_; dashed line, FEF_25-75_.

Data available from a program of periodic employment-based spirometric monitoring dating back 16 years prior to presentation, along with nearly five years of subsequent follow-up, are shown in Figure 2. Taking into account 18 serial measurements taken over 241 months, analyzed by least squares regression, the patient's FEV_1 _fell by 77 ml/year, FVC fell by 54 ml/year and FEF_25-75 _fell by 110 ml/s/year overall. Using National Institute for Occupational Safety and Health (NIOSH) Spirometry Longitudinal Data Analysis (SPIROLA) software for analysis of lung function time trends [6], the change in FEV_1 _measured during the period of employment (-128 ml/year) demonstrated an accelerated decline relative to the projected normal age-related decrease of FEV_1 _(-42 ml/year) [7]. The trajectory of plotted lung function crosses below the age-based 95th percentile lower limit of normal (LLN) approximately midway in this occupational exposure period. After the patient's retirement, his FEV_1 _remained below the LLN, but the slope of its decline (46 ml/year) shifted closer to the normal age-related trajectory. The within-person variation in FEV_1 _remained within the SPIROLA-based normal range. The overall slope of the FVC was steeper than the normal age-predicted decline, but only crossed the LLN later in follow-up.

To further assess the pattern of change over time, we remodeled the data in Figure 2 in a "break-point" analysis, jointly estimating linear regression parameter estimates for lung function decline over the periods before and after the patient's retirement. For FEV_1_, the parameter estimate before retirement was negative (in the direction of loss) and significant (*P *< 0.05), but positive, albeit non-significantly, after his retirement (*P *= 0.7). For FVC, the parameter estimate for the period before the patient's retirement was close to zero (*P *> 0.99), and post-retirement the parameter estimate deflected negatively (*P *< 0.05). For FEF_25-75_, the pre-retirement parameter estimate was significantly negative and then positive after the breakpoint (*P *< 0.01 for each).

A repeat CT scan of the chest after almost five years of follow-up showed progression of panlobular severe emphysema and areas of scarring in the bilateral bases (not shown). Repeat pulmonary function testing demonstrated a TLC volume by plethysmography of 6.37 litres (94% of the predicted value), RV of 3.10 litres (126% of the predicted value), a RV:TLC ratio of 49%, and D_L_CO adjusted for alveolar volume of 2.8 (63% of the predicted value). To date, the patient has not wished to initiate A1AT replacement therapy. The patient was recognized by his employer's workers' compensation carrier as having COPD attributable in part to his occupational exposure, along with co-attribution to non-work-related factors (A1AT deficiency and cigarette smoking).

## Conclusion

This patient presented with symptomatic airflow obstruction and emphysema in the context of underlying A1AT deficiency. Importantly, the patient manifested accelerated lung function decline during the period of highest occupational concrete dust exposure and attenuated decline once such exposure ceased. Using a NIOSH-recommended analytic approach, the decline in FEV_1 _values from workplace surveillance spirometry was found to be steeper than the normal age-associated slope, but attenuated after occupational exposure discontinued. This divergence in trajectory is also consistent with the separate break-point statistical analysis we performed. Moreover, the patient's post-exposure decline in FEV_1 _(-46 ml/year) is slightly less than the annual decline that has been reported in untreated A1AT patients (-60 ± 7 ml/year) [8]. Of note, we do not have systematic data on symptoms over time to correlate with the lung function data; for example, repeated measures using the Borg Dyspnea scale or the Medical Research Council assessment for shortness of breath.

Although cigarette smoking is a well-established co-factor in airflow obstruction and emphysema among people with A1AT deficiency, occupation has also been implicated as a risk factor for worse disease status in several epidemiological studies of A1AT-deficient people with or without work-related exposures. The largest study of this question found a lower FEV_1 _value in adults ages 50 years and above with PiZZ phenotype A1AT who self-reported occupational exposures to gas, fumes or dust for at least three months [3]. Other cross-sectional observational studies support this association [4,5]. A recent study also reported accelerated decline in lung function among 11 rescue workers with mild to moderate A1AT deficiency (no PiZZ phenotypes) exposed to dust after the World Trade Center collapse [9].

Studies of uncontrolled dry concrete grinding operations have documented very high levels of both respirable suspended particulates and silica dust [10]. In non-A1AT-deficient populations, construction work involving inorganic dust exposures (which subsumes concrete finishing) is associated with increased COPD mortality risk [11], and occupational silica exposure (as noted, an important constituent of concrete dust) is linked to chronic airflow obstruction [12]. The specific pattern of exposure and response in our patient, together with the broader epidemiological evidence [1,2,11,12], is consistent with a relationship between the patient's dust exposure and his COPD, with the acknowledgment that A1AT deficiency was a key mediating factor. Moreover, our report is unique in describing longitudinal exposure history coupled with measured lung function data supporting this potential effect-moderating relationship.

People with PiZZ phenotype A1AT disease should be assessed for occupational exposures and closely monitored for work-accelerated progression of lung function decline over and above the decline associated with this disease absent such exposure. More generally, this case not only points to the biological plausibility of occupationally associated COPD but also underscores that work-associated pulmonary disease can be multi-factorial.

## Abbreviations

A1AT: α_1_-antitrypsin; COPD: chronic obstructive pulmonary disease; FEF_25-75_: forced expiratory flow over 25% to 75% of the expired volume.

## Consent

Written informed consent was obtained from the patient for publication of this case report and any accompanying images. A copy of the written consent is available for review by the Editor-in-Chief of this journal.

## Competing interests

The authors declare that they have no competing interests.

## Authors' contributions

MZ was a major contributor in writing the manuscript. PQ and PB were involved in analyzing the data and were minor contributors in writing the manuscript. All authors read and approved the final manuscript.
